# Evolution of Public Knowledge About Dementia Causes and Symptoms: A Gender Perspective

**DOI:** 10.1093/geroni/igaa057.184

**Published:** 2020-12-16

**Authors:** Pedro Santos, Constanca Paul, Cláudia Silva

**Affiliations:** 1 Biomedical Sciences, Abel Salazar Biomedical Sciences Institute - University of Oporto, Porto, Portugal; 2 University of Porto, Porto, Portugal; 3 ESSA, Lisbon, Portugal

## Abstract

The research objective is to monitor the evolution of public knowledge about dementia causes and symptoms, over a three-year period and by gender. The survey was made available at the Directorate-General of Health website and disseminated by email to relevant health and social stakeholders and through social networks, in 2015 and 2018. Respondents (n=1478 and 1716, respectively), included mostly women (79.4% and 83.3%). In both years, respondents showed a higher knowledge on symptoms than on causes. Total knowledge about symptoms and combined knowledge scores were higher in 2018 compared to 2015 (p=.012 and p=.0.2), respectively). “Neurological brain changes" were considered the main causes of dementia, by both genders in 2015 and in 2018 (>80% of respondents), with an increase in relative frequency being observed only for women (p=.039). “Psychiatric disease” and "drug consumption" are now less regarded as causes of dementia by both genders, with significant change over time also among women (p=.006 p=.001). On the contrary, in the last survey more women (+3.7%; p=.049) and men (+9.3%; p=.022) considered “stress” as main cause of dementia. “Confusion and disorientation”, “wandering and getting lost”, “difficulty managing and paying bills”, ”difficulty remembering things from the day before”, and “difficulty managing daily tasks”, were considered the most common symptoms, but only the last two significantly increased in 2018 (p=.018 and p=.000). Women knowledge increased regarding more causes and more symptoms compared to men. These findings will help to inform public debate and decision-making on gender-based policies to address awareness and stigma about dementia.

